# Engineering of Injectable Antibiotic-laden Fibrous Microparticles Gelatin Methacryloyl Hydrogel for Endodontic Infection Ablation

**DOI:** 10.3390/ijms23020971

**Published:** 2022-01-16

**Authors:** Juliana S. Ribeiro, Eliseu A. Münchow, Ester A. F. Bordini, Nathalie S. Rodrigues, Nileshkumar Dubey, Hajime Sasaki, John C. Fenno, Steven Schwendeman, Marco C. Bottino

**Affiliations:** 1 Department of Cariology, Restorative Sciences, and Endodontics, School of Dentistry, University of Michigan, Ann Arbor, MI 48104, USA; sribeirooj@gmail.com (J.S.R.); esterbordini@gmail.com (E.A.F.B.); nathalie.sard@gmail.com (N.S.R.); nileshd@nus.edu.sg (N.D.); hajimes@umich.edu (H.S.); 2 Department of Restorative Dentistry, School of Dentistry, Federal University of Pelotas, Pelotas 96015-560, Rio Grande do Sul, Brazil; 3 Department of Conservative Dentistry, School of Dentistry, Federal University of Rio Grande do Sul, Porto Alegre 90035-003, Rio Grande do Sul, Brazil; eliseumunchow@gmail.com; 4 Department of Dental Materials and Prosthodontics, School of Dentistry, São Paulo State University, Araraquara 14801, São Paulo, Brazil; 5 Department of Biologic and Materials Sciences & Prosthodontics, University of Michigan School of Dentistry, Ann Arbor, MI 48104, USA; fenno@umich.edu; 6 Department of Pharmaceutical Sciences and the Biointerfaces Institute, University of Michigan, Ann Arbor, MI 48104, USA; schwend@med.umich.edu; 7 Department of Biomedical Engineering, College of Engineering, University of Michigan, Ann Arbor, MI 48104, USA

**Keywords:** electrospinning, cryomilling, biodegradation, antibiotics, fibrous particles, regeneration, dentistry, endodontics

## Abstract

This study aimed at engineering cytocompatible and injectable antibiotic-laden fibrous microparticles gelatin methacryloyl (GelMA) hydrogels for endodontic infection ablation. Clindamycin (CLIN) or metronidazole (MET) was added to a polymer solution and electrospun into fibrous mats, which were processed via cryomilling to obtain CLIN- or MET-laden fibrous microparticles. Then, GelMA was modified with CLIN- or MET-laden microparticles or by using equal amounts of each set of fibrous microparticles. Morphological characterization of electrospun fibers and cryomilled particles was performed via scanning electron microscopy (SEM). The experimental hydrogels were further examined for swelling, degradation, and toxicity to dental stem cells, as well as antimicrobial action against endodontic pathogens (agar diffusion) and biofilm inhibition, evaluated both quantitatively (CFU/mL) and qualitatively via confocal laser scanning microscopy (CLSM) and SEM. Data were analyzed using ANOVA and Tukey’s test (α = 0.05). The modification of GelMA with antibiotic-laden fibrous microparticles increased the hydrogel swelling ratio and degradation rate. Cell viability was slightly reduced, although without any significant toxicity (cell viability > 50%). All hydrogels containing antibiotic-laden fibrous microparticles displayed antibiofilm effects, with the dentin substrate showing nearly complete elimination of viable bacteria. Altogether, our findings suggest that the engineered injectable antibiotic-laden fibrous microparticles hydrogels hold clinical prospects for endodontic infection ablation.

## 1. Introduction

The success of endodontic regenerative treatment depends on the elimination of intraradicular microorganisms and the establishment of a microenvironment favorable to the proliferation and differentiation of stem cells [1]. Over decades, calcium hydroxide was the antimicrobial agent most commonly used for the disinfection of contaminated root canals [2], but its effectiveness was revealed to be limited against some pathogens, such as *Enterococcus faecalis* (*E. faecalis)*, *Actinomyces naeslundii* (*A. naeslundii*), and *Candida albicans* (*C. albicans*) [3,4]. Moreover, the overall efficacy of calcium hydroxide within the dentinal tubules seemed unreliable [5]. Thus, other intracanal medicaments (e.g., antibiotics) or the combination of different agents seemed paramount to maximizing the eradication of microorganisms [6,7]. Notwithstanding, the local administration of antibiotics may result in some negative side effects (e.g., bacterial resistance, cell toxicity, among others) [8,9,10], making it relevant to the continued search for an effective and biologically safe drug delivery method capable of penetrating the root canal system of infected pulp tissues.

In light of offering a biocompatible scenario for the disinfection of contaminated root canals, biodegradable drug delivery systems (e.g., scaffolds and hydrogels) laden with antimicrobial agents have gained the attention of researchers, and several studies are currently characterizing their clinical potential [11,12,13,14]. Concerning the use of fibrous scaffolds, electrospinning is a straightforward method for synthesizing antibiotic-laden polymeric nanofibers [6,9,15,16]. Notably, a tubular 3D construct based on triple antibiotic-eluting fibers has already been developed to fit within the intracanal space of infected teeth [6]; thus, allowing significant antimicrobial activity, the elimination of bacterial biofilm inside dentinal tubules, and more importantly, healing of damaged periapical tissues [6]. Nevertheless, electrospun fibers may not be capable of complete eradication of root canal infections, especially due to the complex anatomical geometry of the root canal system (e.g., the existence of lateral canals and apical ramifications), which may be difficult to access by a solid drug-releasing approach such as the 3D construct described earlier. In order to improve the bioavailability of antimicrobial agents into the entire root canal system, the incorporation of electrospun fibers into hydrogels has also been attempted, showing promising results due to more controlled degradation and drug release profile [17,18,19]. Despite effectiveness, the strategy of combining electrospun fibers with a hydrogel may present some drawbacks, such as inadequate dispersion of the fibers within the hydrogel matrix. Alternatively, enhanced miscibility between nanofibers and the hydrogel matrix was demonstrated when electrospun fibers were further processed via cryomilling into microspheres or small-sized particles [20,21]. Relevant to clinical dentistry, gelatin methacryloyl (GelMA) is a semi-synthetic biocompatible and biodegradable hydrogel with interesting structural characteristics, such as curing capacity, stability at physiological temperature, and cell-friendly behavior [22]. Of note, a recent study by Monteiro et al. [23] demonstrated that GelMA can be easily photopolymerized using a dental curing light, representing a promising method of placing injectable biomaterials into the root canal system following a chairside procedure. Considering that no previous attempt has been made to engineer injectable antibiotic-laden fibrous microparticles GelMA hydrogels, herein, electrospun fibers loaded with clindamycin (CLIN) or metronidazole (MET) were effectively processed into antibiotic-laden microparticles and successfully used to modify a well-known photo-curable gelatin methacryloyl hydrogel, guaranteeing excellent biological and antimicrobial/antibiofilm properties to fight endodontic infections. Altogether, our findings suggest that the engineered injectable antibiotic-laden fibrous microparticles hydrogels hold clinical prospects for endodontic infection ablation.

## 2. Results

### 2.1. Antibiotic-laden Fibers and Cryomilled Fibrous Microparticles

**Figure 1:**
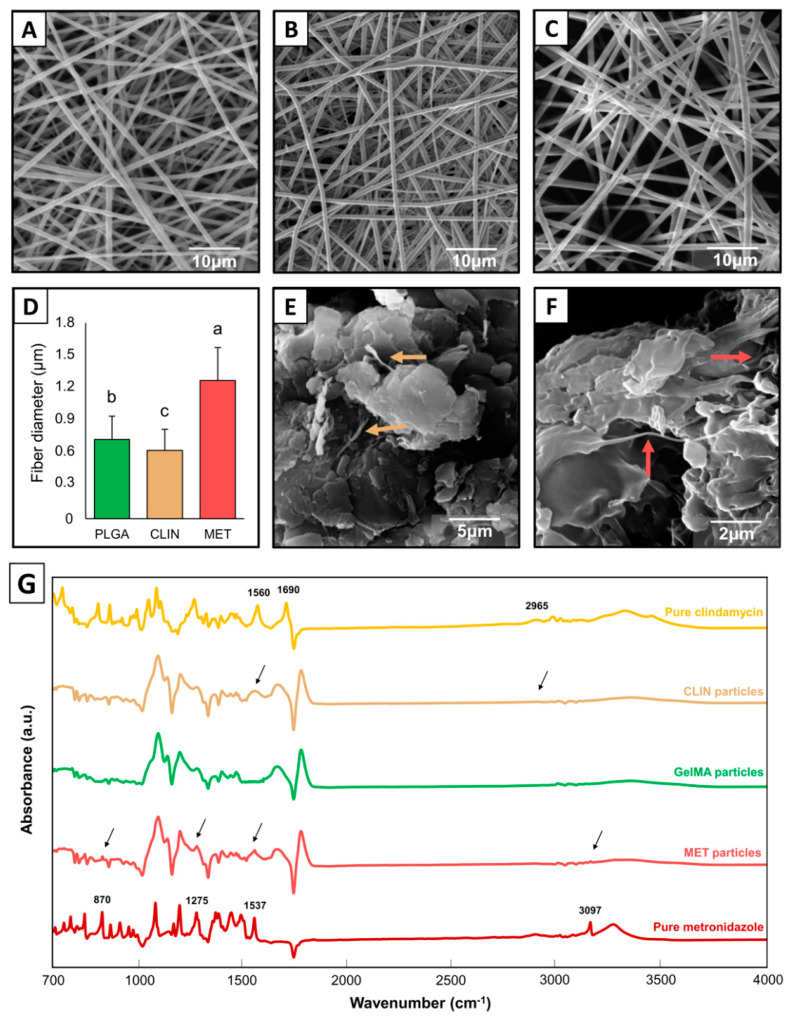
Representative SEM micrographs of electrospun and cryomilled fibers. (**A**) PLGA fibers (control); (**B**) CLIN-laden fibers; (**C**) MET-laden fibers; (**D**) graph showing the average fiber diameter of the electrospun fibers, with different letters above standard deviation bars, indicating statistical differences among the groups (*p* < 0.05); (**E**) fiber-based particles comprised of CLIN, yellow arrows indicate CLIN-laden fibers, and (**F**) fiber-based particles comprised of MET, red arrows indicate MET-laden fibers; and (**G**) FTIR spectra of pristine antibiotics (CLIN and MET) and GelMA particles, as well as the processed antibiotic-modified GelMA particles, black arrows indicate characteristics peaks of each antibiotic. SEM, Scanning Electron Microscope; PLGA, Poly(lactic-co-glycolic acid); CLIN, Clindamycin; MET, metronidazole; FTIR, Fourier-transform infrared spectroscopy; GelMA, gelatin methacryloyl hydrogel.

The SEM micrographs shown in Figure 1 demonstrate a bead-free fiber morphology for the obtained electrospun fiber mats. While the MET-based fibers (Figure 1C) were thicker (*p* < 0.001) than the PLGA fibers (Figure 1A), the CLIN-based fibers (Figure 1B) exhibited a thinner fiber diameter distribution (*p* = 0.042). All the fiber mats showed an average fiber diameter that was statistically different from each other (*p* < 0.001; Figure 1D). SEM micrographs of the cryomilled fibers showed the formation of a uniform set of GelMA-based fiber particles, with both the CLIN- (Figure 1E) and MET-laden (Figure 1F) microparticles demonstrating homogeneous dispersion within the GelMA matrix without any signs of aggregation. Even though some typical peaks of CLIN and MET overlapped with the peaks of GelMA, Figure 1G reveals that both antibiotics were successfully incorporated within the GelMA particles. The peaks at 1560 cm^−1^ (C=C), 1690 cm^−1^ (C=O), and 1965 cm^−1^ (C–H) are related to the stretching vibration of CLIN, [24] whereas the peaks at 870 cm^−1^ (C–NO_2_), 1275 cm^−1^ (C–O), 1537 cm^−1^ (N=O), and 3097 cm^−1^ (=C–H) are related to the stretching vibration of MET [25,26].

### 2.2. Antibiotic-laden Fibrous Microparticles in Gelatin Methacryloyl Hydrogels

#### 2.2.1. Swelling and Biodegradation

**Figure 2:**
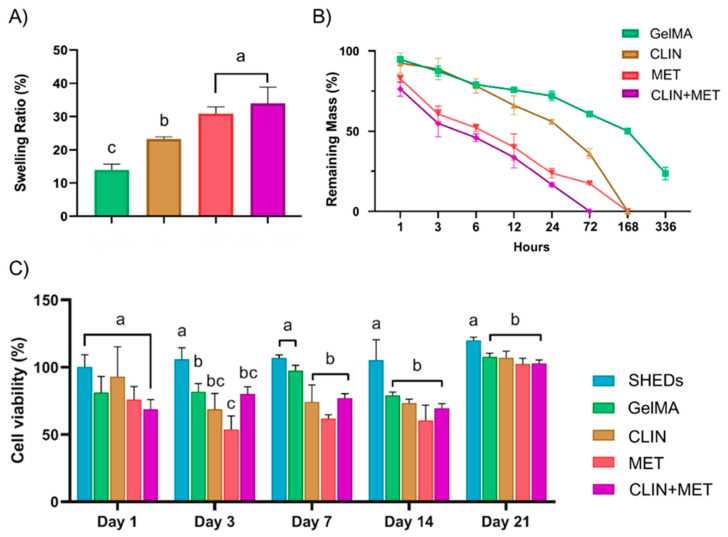
Graphs showing the swelling ratio (**A**), the degradation profile (**B**), and the cell viability (**C**) of the engineered hydrogels. The swelling ratio (%) results indicate the amount of water absorbed by the hydrogels within a 24 h period at 37 °C. The results for the in vitro degradation test reveal the mass loss of all hydrogels after exposure to DI water containing 1 U/mL of collagenase type I at 37 °C for 336 h. The results for the viability of stem cells from exfoliated deciduous teeth (SHEDs) were obtained indirectly using an MTS assay after 24 h of cell exposure in response to aliquots of the hydrogels at days 1, 3, 7, 14, and 21. The percentage of cell viability was normalized by the mean absorbance of SHEDs cultured at day 1 (100%). All the results are presented as mean ± SD values (*n* = 4/group). Distinct letters above the standard deviation bars indicate statistically significant differences among the groups (*p* < 0.05). GelMA, gelatin methacryloyl hydrogel; CLIN, Clindamycin; MET, metronidazole.

Figure 2 shows the swelling ratio (A), degradation profile (B), and cell viability (C) of the synthesized hydrogels. Modification of GelMA with the antibiotic-laden cryomilled particles significantly increased the swelling ratio of the hydrogel (*p* < 0.05), ranging from 15.4% in the fiber-free GelMA to 37% in the CLIN+MET-based GelMA. The hydrogels incorporated with MET-laden fiber particles (MET and CLIN+MET groups) resulted in a greater swelling ratio than the CLIN-based hydrogel (*p* < 0.05). All hydrogels displayed a degradation profile starting within the first hour of the experiment, although the groups comprised of MET-based particles seemed to produce a faster and more intense degradation profile as compared with the CLIN and GelMA groups. CLIN+MET hydrogel lost nearly 50% of the initial mass after 6 h, and at the 72 h time point, it was degraded. CLIN- and MET-based hydrogels showed a slower extent of degradation than the CLIN+MET group, with complete degradation of the former occurring at the 168 h time point. The fiber-free GelMA was not completely degraded after 336 h, showing approximately 20% of the remaining mass after enzymatic degradation.

#### 2.2.2. Cell Viability

Concerning cytocompatibility of the tested hydrogels, a reduction in SHEDs’ viability was observed at every time point investigated, especially for the materials containing MET. However, at day 1 there was no statistical difference between the groups and the control (SHEDs) (*p* > 0.05), although, at day 3, all groups showed a cell viability potential that was statistically lower (*p* < 0.05) than the control and below the 80% level. The antibiotic-laden fibrous microparticle hydrogels sustained a lower cytocompatibility compared with the control at days 7 and 14, although without any cytotoxic behavior, ranging from 61% to 70% of cell viability. All hydrogels presented cell viability of ~100% when testing aliquots collected at day 21.

#### 2.2.3. Antimicrobial Efficacy

**Figure 3:**
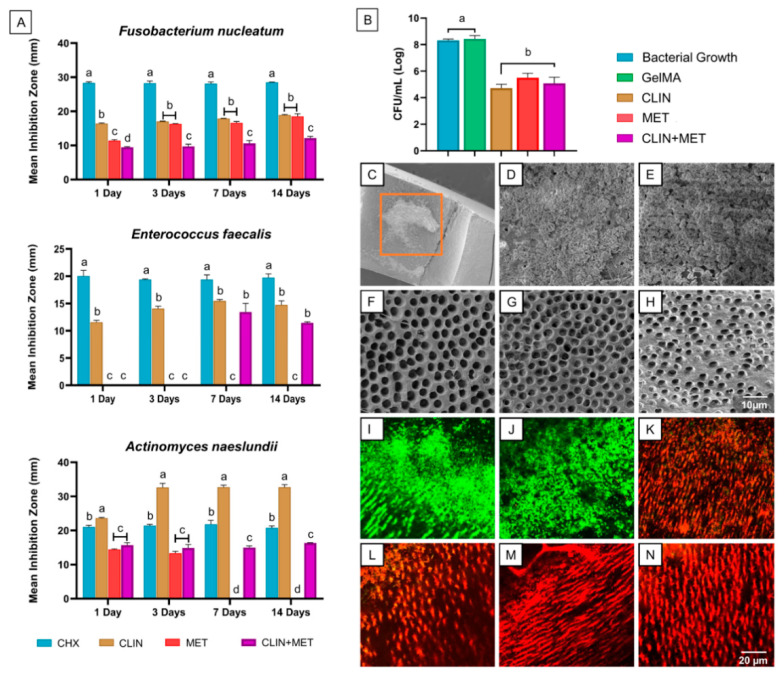
Results for the antimicrobial properties of the tested hydrogels. (**A**) Graphs showing the results (mean inhibition zones, in mm) from the agar diffusion assays against three bacteria at days 1, 3, 7, and 14. Chlorhexidine (CHX) served as the positive control. (**B**) Graph depicting the results (mean counts of colony-forming unit [CFU/mL]) from the *A. naeslundii* biofilm model used in the study, having a negative control group consisting of untreated bacterial growth. Distinct letters above the standard deviation bars indicate statistically significant differences among the groups (*p* < 0.05). (**C**) Representative SEM micrograph showing the evaluated areas of each sample (inner root walls of dentin slices). (**D**–**H**) SEM micrographs for the negative control (**D**) and groups treated with GelMA (**E**), CLIN-based hydrogel (**F**), MET-based hydrogel (**G**), and CLIN+MET-based hydrogel (**H**). (**I**–**N**) CLSM micrographs of 7-day *A. naeslundii* biofilm imaged from inner root canal walls. Images are related to the negative control group (**I**), antibiotic-free GelMA (**J**), CLIN-based hydrogel (**K**), MET-based hydrogel (**L**), CLIN+MET-based hydrogel (**M**), and 2.5% sodium hypochlorite (**N**), which served as the positive control. CLSM images were collected in sequential illumination mode by using 488 nm and 552 nm laser lines. Fluorescent emission was collected in 2 HyD spectral detectors with filter range set up to 500–550 nm and 590–655 nm for green (SYTO9) and red dye (PI), respectively. SEM, Scanning Electron Microscope; GelMA, gelatin methacryloyl hydrogel; CLIN, Clindamycin; MET, metronidazole; CLSM, confocal laser scanning microscopy.

Results for the antimicrobial properties (agar diffusion assay) of the antibiotic-laden fibrous microparticles hydrogels are shown in Figure 3A. All hydrogels showed antimicrobial action against the bacteria, except for MET against *E. faecalis* at all periods tested and against A. naeslundii at days 7 and 14; and the CLIN+MET group when tested against *E. faecalis* at days 1 and 3, which did not display any inhibition potential. The antibiotic-modified hydrogels showed overall lower antimicrobial activity compared with the control (CHX), except when tested against A. naeslundii, in which CLIN resulted in greater inhibition zones (*p* < 0.05). Among the hydrogel groups, CLIN demonstrated increased antimicrobial effectiveness, especially against A. naeslundii. MET was as effective as CLIN when tested against *F. nucleatum*, although its antimicrobial action against A. naeslundii was limited to the first 3 days, and against *E. faecalis*, it presented no inhibition potential. The CLIN+MET-laden hydrogel overall exhibited lesser effectiveness compared with the isolated counterparts (*p* < 0.05), and its antimicrobial activity against *E. faecalis* was time-dependent, resulting in inhibition zones only after day 7.

The antibiotic-free hydrogel (GelMA) and the negative control group (bacterial growth) neither impair biofilm formation nor reduce cells viability, as demonstrated by high colony-forming units’ (log_10_ CFU/mL) values (Figure 3B). Meanwhile, all antibiotic-modified hydrogels showed antibiofilm effects, resulting in lower CFU counts than the controls (*p* < 0.05). The antibiofilm activity was similar among the modified hydrogels, regardless of the type or combination of antibiotics (*p* > 0.05). SEM micrographs shown in Figure 3 reveal a homogeneous accumulation of biofilm for the negative control (Figure 3D) and GelMA (Figure 3E) groups after 7 days of A. naeslundii culture on dentin. Conversely, dentin treated with the antibiotic-modified hydrogels showed the absence of biofilm (Figure 3F–H), with the dentin tubules apparently being empty. As verified in the CLSM micrographs related to the negative control and GelMA groups (Figure 3I,J), a dense population of viable bacteria attached to the dentin surface and penetrating dentinal tubules was identified. However, after the application of the antibiotic-modified hydrogels, the dentin substrate showed nearly complete elimination of viable bacteria (Figure 3K–M), similar to the positive control (Figure 3N).

## 3. Discussion

The drug delivery system developed in this study combined the effects of two straightforward releasing vehicles (i.e., electrospun fibers and fiber-based particles) to obtain a hybrid mechanism for the safe and sustained release of antibiotics targeting the elimination of root canal infections. More importantly, our main goal was to offer a drug delivery system in the form of an injectable and photo-curable hydrogel, aiming to establish a feasible chairside procedure of easy application with effective antimicrobial action.

The fibers synthesized via electrospinning were all morphologically adequate, showing a smooth and homogeneous fiber diameter distribution. The CLIN-based fibers presented the thinnest average fiber diameter of 628 nm (±194 nm), which was lower than the PLGA fibers (729 ± 214 nm) and the MET-based fibers (1.3 ± 0.3 µm). Here, both antibiotics (CLIN and MET) possess a hydrophilic nature [27], which may decrease the viscosity of the polymer solution, allowing for the formation of thinner fibers [28]. However, the MET-based fibers did not show such thin distribution, exhibiting ca. two-fold higher thickness values, probably due to the single hydroxyl group found in MET, which may have increased hydrogen bonding interactions and cross-linking of the polymer network, thereby reducing spinnability during electrospinning and the acquisition of thicker fibers [28]. Overall, the incorporation of antibiotics did not compromise morphological features of the electrospun fibers, which is an essential aspect for their proper functioning as a drug-releasing vehicle.

Considering that electrospun fibers may result in the burst release of drugs during the first 24 h [16,27,29], we have further processed them into small-sized particles, aiming to obtain a more controlled and sustained release of CLIN/MET. The method used here was cryomilling, which consists of cooling the material, then reducing it to smaller particles. According to some studies [29,30], this approach can maintain or even improve the therapeutic properties of the original fibers. The matrix used for the embedment of the fibers prior to cryomilling was GelMA (i.e., the same polymer used to prepare the injectable hydrogel system) so that better distribution and chemical compatibility between the fiber-based particles and the hydrogel could be expected without affecting hydrogel injectability. Considering that the incorporation of CLIN and MET into the respective hydrogels was confirmed by the identification of typical FTIR peaks (Figure 1G), we anticipated that the engineered antibiotic-laden fibrous microparticles hydrogels would demonstrate antimicrobial properties.

The release rate of any drug or therapeutic compound relies on the degradation speed of the carrying vehicle [12,22]. In the case of GelMA, the degradation speed is inversely correlated to three main factors: the degree of functionalization of the compound, the concentration of the hydrogel, and the total amount of enzymes [31]. The foregoing characteristics were all kept constant in our study. In light of verifying the degradation ability of the hydrogels, we performed two different tests: hygroscopic swelling and in vitro enzymatic degradation. As shown in Figure 2A, the antibiotic-modified hydrogels absorbed a greater amount of humidity (PBS) when compared with the neat GelMA, probably due to the hydrophilic nature of CLIN and MET [27]. Both of these antibiotics are comprised of hydroxyl groups, hence increasing polar interactions and the formation of hydrogen bonding. Nevertheless, it may be suggested that MET is more hydrophilic than CLIN due to the more heterogeneous composition of the latter (e.g., elements, such as phosphorus, chlorine, and sulfur). Thus, the presence of MET would make the GelMA matrix swell to a greater extent as compared with the presence of CLIN. No less important, the porosity of the synthesized fibers may have also played a role in the swelling ratio of the fiber-modified hydrogels, since the higher the degree of porosity of the fiber mat, the more intense its hygroscopic behavior [32,33]. Despite the fact that we did not conduct any analysis to determine the porosity level of the fiber mats, one should note that the MET-laden fibers showed the thickest morphology and the most porous fiber architecture. Thus, the total amount of GelMA matrix embedded within the fiber mat was also probably higher, turning the MET fibrous particles more prone to hydrolysis [27]. This may explain the more intense hygroscopic swelling and biodegradation patterns demonstrated by the MET and CLIN+MET hydrogels when compared with the CLIN group.

From the findings presented here, one can note that our antibiotic-modified hydrogels would work adequately since they demonstrated a one-week driven degradation profile, which is indeed desirable for a dressing medication that aims to eliminate root intracanal infection during a clinical inter-appointment period. It is the sustained release of antibiotics during that one-week interval that could effectively act in the ablation of root canal infections. However, it is paramount that the release of antibiotics does not reach cytotoxic levels; otherwise, the hydrogels would interfere with tissue healing/regeneration events, thus impairing the clinical translation of our strategy. According to the results shown in Figure 2C, the hydrogels incorporated with the antibiotic-laden fibrous particles did not exhibit any significant cytotoxic effects on SHEDs. Even so, one may note that lower cell viability was identified for the antibiotic-modified hydrogels at days 3 and 7, probably due to the release of CLIN and MET during degradation of the GelMA matrix. Even though antibiotics are usually cytotoxic when administered at high concentration levels [34], the amount of CLIN and MET used in our study was minimal, so their release would not be responsible for a significant reduction in the viability of SHEDs. Here, one would suggest that cell proliferation into the photopolymerized hydrogel may occur at a slower rate of speed, increasing once the moderately cross-linked structure of the hydrogel starts to degrade. Of note, cell viability was importantly increased after complete degradation of the hydrogels, suggesting that any byproducts originated during degradation and the release of CLIN and MET did not cause toxic effects to the cells. Last but not least, all the experimental hydrogels demonstrated cell viability values above the cytotoxicity threshold of 50%, suggesting their clinical safety and suitability.

In light of verifying the antimicrobial properties of the experimental hydrogels, we conducted two distinct antimicrobial analyses: agar diffusion and biofilm inhibition assays. Concerning the first analysis, the CLIN-based hydrogel was the only GelMA material showing antimicrobial activity against all the bacteria species at every time point investigated in the study (Figure 3A), corroborating to the findings of a previous study [35], which showed that microorganisms are usually highly susceptible to CLIN but not always to MET. Here, three bacteria (*F. nucleatum*, *E. faecalis*, and *A. naeslundii*) were considered in the agar diffusion assay due to their broad association to cases of infected immature teeth and failed endodontic treatment with persistent infection [36,37]. The foregoing bacteria are usually pathogenic and difficult to eliminate, but as verified by our findings, the strategy of using a hydrogel system incorporated with antibiotics resulted in important inhibition values, although it was dependent on the type of antibiotic(s) used, as well as the type of bacteria tested. While CLIN is typically a bacteriostatic agent, acting on the inhibition of bacterial protein synthesis [27], MET is a bactericidal antimicrobial competing with the biological electron acceptors of bacteria, disturbing their energy metabolism, and thus causing cell death [27]. Having this in mind, MET was expected to be less effective against Gram-positive (G+) bacteria, which presents a more organized cell wall structure, compared with the Gram-negative (G–) counterparts. Indeed, MET was effective when tested against *F. nucleatum* (G–), but it did not result in any inhibition potential against *E. faecalis* (G+). It seems that the concentration of MET released through the present drug-delivery strategy was insufficient for the proper growth inhibition of G+ bacteria. Conversely, CLIN was effective against all three bacteria, and especially to *A. naeslundii* (G+), which suggests that this bacterial species is a very sensitive microorganism to CLIN. Indeed, CLIN may act as a direct peptidyltransferase inhibitor in the case of sensitive microorganisms, hence affecting the process of the peptide chain initiation and stimulating dissociation of peptidyl-tRNA from ribosomes, i.e., a potent antibacterial inhibition mechanism [38].

In our study, one hydrogel was prepared by mixing equal amounts of the CLIN- and MET-laden fibrous microparticles to elucidate whether the antimicrobial activity would be potentiated upon the presence of both antibiotics into the same hydrogel. Nevertheless, the inhibition potential was not amplified, indicating the existence of a minimum inhibition concentration level for each of the drugs. One should note that the concentration of each antibiotic released from the CLIN+MET hydrogel was probably lower than the concentrations derived from the single-mix CLIN and MET groups; therefore, explaining the overall lower or lack of statistical differences in the antimicrobial results of those groups. Remarkably, the MET hydrogel did not result in consistent antimicrobial activity to all of the tested conditions, although the hydrogel modified with the CLIN-laden fibrous microparticles exhibited a steadier antimicrobial action, thus supporting further investigations in pre-clinical animal models of periapical disease. It is noteworthy that on one hand, we could increase the concentration level of MET incorporated into the fibrous particles, aiming to obtain a more significant gain in antimicrobial effect, but on the other hand, we could negatively increase the cytotoxicity of the resultant hydrogel, thus limiting the applicability of MET when using the same strategy. Last, the presence of MET fibrous particles in the CLIN+MET hydrogel formulation allowed a faster GelMA degradation, with the complete breakdown occurring at 72 h of incubation, i.e., a shorter period as compared with the one-week profile shown by the single-mix counterparts, which, as discussed earlier, would be more desirable as an intracanal dressing.

Concerning the biofilm inhibition assay evaluated in this study, SEM and CLSM analyses (Figure 3) revealed almost complete removal of the *A. naeslundii* biofilm upon exposure to our antibiotic-laden fibrous microparticles GelMA, indicating that this hybrid strategy is effective in the ablation of even complex structures such as a highly-organized biofilm. Different from the negative control (bacterial growth) and antibiotic-free GelMA groups, the hydrogels containing CLIN- and MET-laden microparticles significantly decreased the counts of viable bacteria in our *A. naeslundii* biofilm model, showing that the fiber-particle vehicle proposed here would provide a satisfactory diffusion of antibiotics through dentinal tubules, as well as to the entire extent of the root canal system (e.g., lateral canals and apical ramifications). Even though our study used a standard sample of human radicular dentin, rather than a full root canal model, the present methodology is commonly used due to its ability to mimic the clinical scenario as it facilitates the continuous formation of biofilms and penetration of bacteria within dentinal tubules [39].

The great novelty of the present study relates to the fact that the antimicrobial mechanisms of small-sized particles are still complex to understand due to the variety of mechanisms involved, making it difficult for bacterial cells to become resistant [40]. This highlights the importance of our strategy, which combines the beneficial effects of small-sized fibers and particles to that of the minimum use of antibiotics to combat bacterial infection, consisting of a biocompatible and therapeutic effective drug delivery approach with minimal possibility of causing bacterial resistance. Moreover, the release of small-sized compounds, such as the antibiotic-loaded fibrous particles synthesized here, may improve the availability of antibiotics at sites of difficult access within the root canal system, perhaps contributing to a more efficacious treatment. To the best of our knowledge, this is the first study that has loaded PLGA electrospun fibers with CLIN and MET and has further processed the fibers via cryomilling in order to obtain microparticles with the drug-releasing ability and the capability of being encapsulated into a photo-curable GelMA hydrogel.

## 4. Materials and Methods

### 4.1. Reagents

Poly(DL-lactide-co-glycolide) (PLGA [75:25], Mw = 97,100, [η] = 0.55–0.75 dL/g, Lactel Absorbable Polymers, Birmingham, AL, USA) pellets, metronidazole (MET), type A gelatin (300 bloom from porcine skin), and methacrylic anhydride were bought from Sigma-Aldrich (St. Louis, MO, USA). Clindamycin phosphate 97.0%+ (CLIN) and lithium phenyl-2,4,6-trimethylbenzoylphosphinate (LAP) were obtained from TCI America Inc. (Portland, OR, USA). Chloroform and methanol were procured from Thermo Fisher Scientific (Waltham, MA, USA), whereas Dulbecco’s phosphate-buffered saline (DPBS) was purchased from Gibco Invitrogen Corporation (Grand Island, NY, USA). All the reagents were used without further purification.

### 4.2. Synthesis of Antibiotic-Releasing Fibers

Three stock polymer solutions were prepared by dissolving PLGA in chloroform to produce an 18 wt.% solution, which was incorporated with different antibiotic mixtures (CLIN or MET dissolved in methanol) at a 15 wt.% concentration relative to the total polymer weight. One stock solution was not incorporated with antibiotics, serving as the control (PLGA). The solutions were stirred overnight, loaded into 5 mL plastic syringes (Becton, Dickson and Company, Franklin Lakes, NJ, USA) fitted with a 27 G metallic needle (Small Parts, Inc., Miami, FL, USA), and then processed via electrospinning using the following parameters: rotating mandrel (120 rpm of speed), a flow rate of 1–2 mL/h, a spinning distance of 20 cm, and 18 kV. The obtained fiber mats were dried overnight at room temperature to remove any residual solvent and stored at 4 °C until use. The morphology and architecture of the fiber mats were analyzed via scanning electron microscopy (SEM; JSM-6390, JEOL, Tokyo, Japan). Samples obtained from each group were mounted on Al stubs and sputter-coated with gold before imaging. The average fiber diameter of 100 single fibers was measured with ImageJ (National Institutes of Health, Bethesda, MD, USA) and expressed as mean ± SD values (in µm).

### 4.3. Preparation of Fiber-Based Particles and Morpho-Chemical Characterizations

The antibiotic-laden fiber mats were processed into fine, small particles via cryomilling [29]. First, the fibrous mats were gently soaked in GelMA, which was synthesized as described elsewhere [22,41]. Briefly, gelatin was solubilized in DPBS at 50 °C to produce a 15 wt.% solution, followed by the dropwise addition of 8 mL of methacrylic anhydride. After 2 h of stirring, 8 mL of DPBS was added at 40 °C to interrupt the reaction, followed by dialysis in deionized water for 1 week to remove salts and unreacted monomers. After hydrogel (GelMA) synthesis, each electrospun fiber mat was cut into small pieces and completely soaked in GelMA. A photocrosslinker (LAP) was added to the mixture at the 0.05% level and stirring (240 rpm) was conducted at 50 °C; crosslinking was achieved for 60 s using a light-emitting diode (LED) curing unit (Bluephase; Ivoclar-Vivadent, Amherst, NY, USA) at 385 nm, resulting in GelMA/fibrous mat samples with a 50 wt.% fiber content. The resin-fiber samples were left to dry in a fume hood overnight, then placed in appropriate metallic milling vials, precooled for 2 min in liquid nitrogen, and milled for 15 min utilizing a cryogenic impact mill (model SPEX CertiPrep 6750, SPEX CertiPrep, Metuchen, NJ, USA). The cryomilling process included alternating 1 min milling cycles separated by 1 min cooling intervals, respectively. The obtained fiber-based particles were stored in a desiccator containing silica at room temperature until further use. SEM imaging was done to verify the morphology of the obtained particles. Fourier-transform infrared spectroscopy (FTIR; Thermo Fischer Scientific, Inc.) was utilized in attenuated total reflection mode ranging between 700–4000 cm^−1^ at a resolution of 4 cm^−1^ to confirm the chemical characteristics of the fiber-based particles incorporated with CLIN and MET.

### 4.4. Fabrication of the Antibiotic-Laden Fibrous Microparticles Gelatin Methacryloyl Hydrogel

The antibiotic-laden fiber particles were sieved (45 μm) before their incorporation into 4 mL of hydrogel solution (15% GelMA), i.e., the same hydrogel synthesized earlier for the GelMA/fiber mats soaking process. Photocrosslinker was added, as described before, followed by the addition of the fiber-based particles at a 5% (*w/v*) level. Four groups were prepared: GelMA—fiber-free GelMA (control); CLIN—GelMA comprised of CLIN-laden fiber particles; MET—GelMA comprised of MET-laden fiber particles; and CLIN+MET—GelMA comprised of equal amounts of CLIN- and MET-laden fiber particles.

### 4.5. Characterization Analyses

Fiber-modified and fiber-free GelMA mixtures were prepared to obtain distinct hydrogel-based samples for the analyses described below. To that end, 100 µL of each mixture was placed into an elastomeric (CutterSil Putty PLUS; Kulzer Dental North America, South Bend, IN, USA) mold of varying dimensions (depending on the test) and irradiated (photopolymerized) for 15 s with the LED at 385 nm.

#### 4.5.1. Swelling and Biodegradation

Cylindrical specimens (*n* = 3/group) were immersed in PBS to allow for swelling for 24 h at 37 °C. Next, the specimens were weighed to establish their wet weight (Ww), then they were lyophilized and weighed again to establish their dry weight (Wd). The volumetric swelling ratio (in %) of each hydrogel group was calculated using the following equation:

Swelling Ratio = (Ww − Wd)/Wd × 100(1)

The in vitro biodegradation of the hydrogels was carried out by incubating cylindrical specimens (6 mm diameter × 2 mm thick) of each hydrogel in an enzymatic solution comprised of DPBS and collagenase type I (1 U/mL, Roche Holding AG, Basel, Switzerland). The specimens (*n* = 4/group) were weighed at baseline (W0) before their incubation in 5 mL of enzymatic solution for 3 weeks at 37 °C; then, the solution was renewed every 3 days with fresh solution to maintain constant enzyme activity. At present time points, the specimens were removed from the solution, washed twice with sterile DI water, blotted dry with low-lint wipes, and weighed again (Wt). The degradation (in %) of each hydrogel group was calculated using the following equation:

Degradation = (Wt/W0) × 100 (2)

#### 4.5.2. Cell Viability

To determine whether modifying the GelMA hydrogel with antibiotic-laden fiber particles would result in cell toxicity, hydrogel specimens (6 mm in diameter × 2-mm thick) were prepared for an in vitro assay following the International Standards Organization guidelines (ISO, 10993-5). Initially, the specimens (*n* = 5/group) were UV-treated for 30 min on each side to disinfect them. Then, they were individually placed in sterile scintillation glass vials (VWR International, LLC, Radnor, PA, USA) that contained 5 mL of alpha-modified Eagle’s Medium (α-MEM; Gibco Invitrogen Corporation, Grand Island, NY, USA) supplemented with 10% fetal bovine serum (FBS; Gibco), L-glutamine (Sigma), 1% penicillin-streptomycin (Gibco), and 1 U/mL of collagenase type I (Roche Holding AG). Next, they were incubated at 37 °C for up to 21 days, with 500 μL aliquots collected at different time points (after 1, 3, 7, 14, and 21 days of storage) to investigate the potential cytotoxic effects of leachable hydrogels’ byproducts (e.g., antibiotics) over time. Equal amounts of storage medium were added back to each vial to maintain a constant extraction volume. Finally, prior to cell exposure, the collected aliquots were filtered through a 0.22 μm membrane (MilliporeSigma, Burlington, MA, USA).

Stem cells derived from human exfoliated deciduous teeth (SHEDs; Lonza, Walkersville, MD, USA) were cultured in an incubator at 37 °C with 5% CO_2_ in α-MEM supplemented with 10% FBS, 1% L-glutamine, and 1% penicillin–streptomycin. Cells at passages 4 to 7 were utilized. SHEDs were seeded at a density of 2.5 × 10^3^ cells/well and were allowed to adhere in the wells of 96-well plates (Corning Incorporated, Corning, NY, USA). After 24 h, the media were subsequently replaced by collected extracts (100 μL) taken from GelMA-based hydrogels. For 24 h, the aliquots were kept in contact with the cells. An amount of 30 μL of CellTiter 96 AQueous One Solution Reagent (Promega Corporation, Madison, WI, USA) was then added to the test wells and allowed to react at 37 °C in a humidified 5% CO_2_ atmosphere. The incorporated dye was measured by reading the absorbance at 490 nm (SpectraMax iD3; Molecular Devices LLC, San Jose, CA, USA) and comparing it with a blank column. SHEDs cultured in complete α-MEM were used as the positive control. Absorbance values were converted to percentages and compared with the test groups’ values.

#### 4.5.3. Antimicrobial Efficacy

The antimicrobial efficacy of the antibiotic-laden fiber-modified hydrogels was verified against endodontic pathogens (agar diffusion) and biofilm inhibition using an infected dentin *A. naeslundii* biofilm model evaluated both quantitatively (CFU/mL) and qualitatively via confocal laser scanning microscopy (CLSM) and SEM. For the agar diffusion assay, the hydrogels were tested against *Actinomyces naeslundii* (*A. naeslundii*, ATCC 12104), *Fusobacterium nucleatum* (*F. nucleatum*, ATCC 25586), and *Enterococcus faecalis* (*E. faecalis*, ATCC 19433) bacteria. Cylindrical-shaped (6 mm diameter × 2 mm thick) specimens were prepared (*n* = 3/group) and disinfected by UV-irradiation (30 min/side). *A. naeslundii* and *F. nucleatum* were anaerobically cultured for 24 h in 5 mL of brain and heart infusion (BHI) broth. *E. faecalis* was aerobically cultured in 5 mL of BHI broth for 24 h. Each bacterial suspension was spectrophotometrically (405 nm) adjusted to obtain 3 × 10^8^ CFU/mL. An amount of 100 μL of each broth was swabbed onto BHI agar plates to form a bacterial lawn. To evaluate the antimicrobial properties over time, GelMA-based hydrogel specimens of the same dimensions were prepared (*n* = 3/group); the specimens were individually incubated in glass vials with 5 mL of sterile PBS for 3 weeks at 37 °C. At predetermined time intervals (1, 3, 7, and 14 days), 500 μL aliquots were drawn and replaced with equivalent amounts of fresh DPBS. The retrieved aliquots were stored at −20 °C until further use. The agar plate was divided into zones: 10 μL of 2% chlorhexidine digluconate (CHX; positive control), 10 μL of DI water (negative control), and 20 µL of GelMA-based aliquots. After incubating for 48 h (*F. nucleatum* and *A. naeslundii*), diameters (in mm) of the clear zones of growth inhibition were measured.

For the anti-biofilm assay, the experiment was conducted only after approval by the local Institutional Review Board (IRB protocol no. 1407656657; University of Michigan). Fifty-four recently extracted, single-rooted human teeth were cleaned and stored in 0.1% thymol until use. The teeth were cut to obtain dentin slices (2 mm thick) so that the crown portion was sectioned 2 mm above the cementum–enamel junction and was cut along the buccolingual plane. The specimens were then wet-finished with SiC papers (600–1200 grit) to obtain both standardized and smooth surfaces, followed by immersion in 2.5% NaOCl and 17% ethylenediaminetetraacetic acid (EDTA) solutions for 5 and 3 min, respectively, under the ultrasonic bath (L&R 2014 Ultrasonic Cleaning System, L&R Ultrasonics, Kearny, NJ, USA). Then, the specimens were rinsed in sterile saline solution for 10 min and autoclaved at 121 °C for 20 min.

*A. naeslundii* (ATCC 12104) was cultivated in an anaerobic chamber for 48 h. The bacterial suspension was adjusted for approximately 7.5 × 10^7^ colony-forming units per milliliter in BHI broth. The sterile dentin slices were placed into 24-well plates that contained 1.8 mL of BHI broth and 0.2 mL of the inoculum and were incubated in an anaerobic chamber for 7 days to allow for the formation of biofilm. The broth was renewed every 2 days. Infected dentin slices (*n* = 6/group) were randomly divided into six groups: GelMA, CLIN, MET, and CLIN+MET hydrogels, as well as Ca(OH)_2_ paste (positive control) and an untreated 7-day-old biofilm (negative control). After 7 days, non-adherent bacteria were removed by gently rinsing the samples in PBS. Next, 50 μL of each material was placed above the biofilm formed and crosslinked for 15 s with the LED. The samples were then incubated for 3 days in the anaerobic chamber and divided for colony forming units (CFU/mL; *n* = 4), scanning electron microscopy (SEM; *n* = 2), and confocal laser scanning microscopy (CLSM; *n* = 3) analyses.

For CFU/mL, the incubated samples were cautiously removed from the wells and placed in Eppendorf tubes containing 1 mL of saline solution. The tubes were sonicated at 30 W for 30 s to detach the biofilms formed on the dentin slices. After that, 100 µL aliquots were collected and subjected to serial dilution, which was carried out in BHI blood agar plates. The plates were then incubated at 37 °C for 24 h in an anaerobic chamber, with the CFU/mL being counted. To prepare them for SEM evaluation, the samples were gently washed in PBS and fixed overnight in 2.5% glutaraldehyde. Next, they were dehydrated in increased concentrations of alcohol/water solutions, treated with increased concentrations of HMDS solutions, and sputter-coated with Au-Pd prior to imaging. For CLSM evaluation, after each treatment was applied, the samples were gently washed with DI water and stained using the fluorescent LIVE/DEAD BacLight Bacterial Viability Kit L-7012 (Molecular Probes, Inc., Eugene, OR, USA). Three areas of each sample were analyzed utilizing 3D reconstruction. They were selected randomly, always starting from the root canal space to the cementum side. A 40× lens (Leica SP2 CL5Mt; Leica Microsystems GmbH, Wetzlar, HE, Germany) was used. The sequence of segments through tissue depth (Z-stacks) was collected using optimal step-size settings (0.35 μm), with the images being composed of 512 × 512 pixels. The excitation-emission maxima for the dyes, respectively, were approximately 480/500 nm for SYTO 9 and 490/635 nm for PI. The images were reconstructed using ImageJ and the percentages of live/dead bacteria were compared with the positive [Ca(OH)_2_] and negative (no treatment) controls to establish statistical significance among the groups.

### 4.6. Statistical Analysis

The obtained data were statistically analyzed (SigmaPlot version 12; Systat Software, Inc., Chicago, IL, USA) using analysis of variance and Tukey’s test for multiple comparisons at the α = 5% level of significance.

## 5. Conclusions

In this work, we successfully designed a photo-curable injectable hydrogel loaded with antibiotic-laden fibrous microparticles, which demonstrated effective antimicrobial activity and non-cytotoxic behavior. Moreover, based on the collected data, the proposed hydrogel holds clinical promise for bacterial infection ablation before regenerative endodontics procedures. Further pre-clinical animal studies (e.g., periapical disease model in rodents) are paramount to investigating the antimicrobial efficacy of this new injectable system.

## Data Availability

The data presented in this study are available on request from the corresponding author.

## Abbreviations

SEM, Scanning Electron Microscope; PLGA, Poly(lactic-co-glycolic acid); CLIN, Clindamycin; MET, metronidazole; FTIR, Fourier-transform infrared spectroscopy; GelMA, gelatin methacryloyl hydrogel; SHEDs, stem cells from exfoliated deciduous teeth; CLSM, confocal laser scanning microscopy; *A. naeslundii*, *Actinomyces naeslundii*; *F. nucleatum*, *Fusobacterium nucleatum*; *E. faecalis*, *Enterococcus faecalis*.
